# Limitations of Only Reporting the Odds Ratio in the Age of Precision Medicine: A Deterministic Simulation Study

**DOI:** 10.3389/fmed.2021.640854

**Published:** 2021-05-14

**Authors:** Avishek Chatterjee, Henry Woodruff, Guangyao Wu, Philippe Lambin

**Affiliations:** The D-Lab, Maastricht University, Maastricht, Netherlands

**Keywords:** precision oncology, precision medicine, odds ratio, risk ratio, prediction model

## Introduction

Precision medicine aims to tailor healthcare to sub-groups of patients identified by common characteristics instead of a one-approach-fits-all paradigm (1–5). By creating prognostic machine learning models based on patient characteristics, we can stratify patients into multiple risk groups, allowing patients in the low(er) risk group to be treated less aggressively, while high(er) risk groups may be treated more aggressively.

When reporting the performance of a binary classifier (such as a machine learning model or a positive/negative lab test with a classification threshold), a pervasive metric in medical literature is the area under the receiver operating characteristic curve (AUROC, or more commonly, AUC) (6, 7), in addition to measures derived from the confusion matrix such as sensitivity, specificity, positive predictive value (PPV), and negative predictive value (NPV).

When assessing the association between a *binary patient characteristic*, say the presence of a comorbidity such as heart disease, and a *binary patient outcome*, say death within a certain follow-up period, a ubiquitous metric in medical literature is the odds ratio (OR) (8). The odds ratio is defined as the ratio of the odds of the outcome in the presence and the absence of that characteristic. The risk ratio (RR), which is the ratio of the probabilities rather than the odds used in the OR, is arguably a more intuitive and useful metric. If the outcome is rare (<<1%), the OR is approximately equal to the RR (9, 10). Several publications exist explaining the rationale for using OR despite its limitations (11–13). The strongest reasons for using OR are: (1) clinical data often makes it impossible to calculate RR, making OR the practical choice, and (2) OR can be easily calculated when using logistic regression.

In this paper, we aim to prove that when using a binary patient characteristic for patient stratification, it is essential to include metrics in addition to OR to provide a complete picture; we do not advocate replacing OR, but supplementing it. While the limitations of OR compared to RR have been discussed in the literature (14–16), in this work, we illustrate their interdependence. Furthermore, using a deterministic simulation, we elucidate the relationship between OR and AUC, providing insight into why reporting OR without AUC can be misleading. We do not suggest that AUC is the most important metric. The reason we chose AUC for performing our simulations is that unlike sensitivity/specificity and PPV/NPV, it is not a paired metric, and thus easy to use in 2-D figures (i.e., OR and AUC), whereas paired metrics would require 3-D figures (e.g., OR, PPV, NPV).

## Description of Simulations

Two experiments were performed. In the first experiment, the simulated patient cohort included *non-exposed* and *exposed* groups, each of size 1,000. Five levels of disease incidence in the non-exposed group (1–5%) were investigated. The RR was varied from 1 to 10 in increments of 0.1. Four cohorts were defined: diseased and non-exposed (DN), healthy and non-exposed (HN), diseased and exposed (DE), healthy and exposed (HE). The odds ratio is then given by:

$$\begin{array}{l} \text{OR}=\left(\text{DE}/\text{DN}\right)/\left(\text{HE}/\text{HN}\right) \end{array}$$

The second experiment was similar to the first, except that the non-exposed group was of size 10,000, and the exposed group was of size 500 (i.e., 20 times smaller than the non-exposed group). In terms of real-world counterparts, the first experiment is similar to a randomized control trial where both the experimental and control arms have 1,000 patients. The second experiment is analogous to a situation where a small percentage of the total patient cohort has a rare condition that impacts patient outcome. In both experiments, the *exposure* in the simulation is naturally binary, rather than a count distribution that has been dichotomized as is the case for Sroka et al. (17). The software used for the simulation was MATLAB R2019a (MathWorks, Natick, MA) and the perfcurve function was used to calculate the AUC, with *exposure* being the feature of interest (0 representing non-exposed and 1 representing exposed).

To demonstrate the negative impact (with respect to predictive modeling) of collecting naturally continuous features as dichotomous, we included a brief study using real world COVID-19 data from a recent publication (18). The training set included 1,810 patients from Bergamo and Pavia; the test set had 381 patients from Rome. We used two features (age and lactate dehydrogenase level during admission) to predict in-hospital mortality. When treating the two features as continuous (like the original publication), we used a Random Forest classifier (with the MaxNumSplits parameter set to 5 to limit overfitting). When treating the two features a binary (age >65 years and LDH >280 U/L), we used a linear discriminant. We compared the AUC of these two models on the test set, using the DeLong test to establish statistical significance.

## Results of Simulation

**Figure 1:**
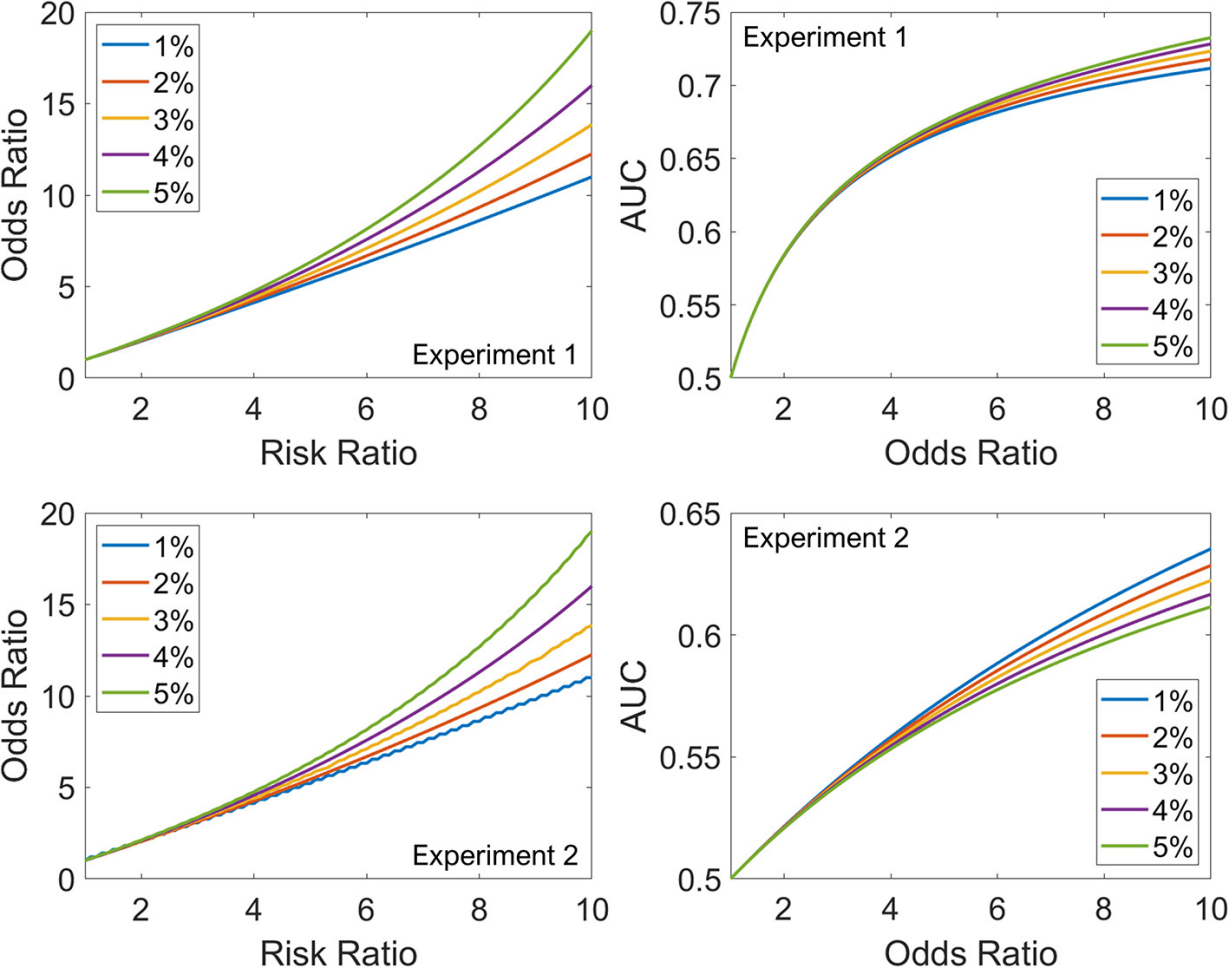
The top row summarizes Experiment 1, and the bottom row summarizes Experiment 2. Left: Relationship between RR and OR as the disease incidence in the non-exposed group increases from 1% (blue) to 5% (green). Right: Relationship between OR and AUC as the disease incidence in the non-exposed group increases from 1% (blue) to 5% (green).

Figure 1 (left panels) shows the relationship between RR and OR as the *disease incidence in the non-exposed group* increases from 1% (blue) to 5% (green) for both experiments. For low values of RR (<3), the five curves overlap, but for higher values of RR, the divergence is striking; at an RR of 10, the OR varies between 11 for 1% disease incidence and 19 for 5%. Irrespective of the ratio of non-exposed and exposed groups (1:1 for Experiment 1 or 20:1 for Experiment 2), the relationship between RR and OR is unaffected, as expected from their definitions. The slight jaggedness in the bottom-left plot is because of rounding effects in the simulation.

Figure 1 (right panels) shows the relationship between OR and AUC as the *disease incidence in the non-exposed group* increases from 1% (blue) to 5% (green). The dependence on disease incidence in the non-exposed group is less marked for AUC-OR relationship than RR-OR relationship. However, unlike the RR-OR relationship, the AUC-OR relationship differs between the experiments. For example, for an OR of 10, the AUC varies between 0.71 and 0.73 in Experiment 1, and between 0.61 and 0.64 in Experiment 2. For the same OR, as exposure becomes less common in the cohort (1:1 for Experiment 1 vs. 20:1 for Experiment 2), the AUC decreases. Also, for the AUC-OR relationship, the order of the curves is different in Experiment 1 (the 1% curve has lower values than the 5%) compared to Experiment 2, where the order is reversed. Thus, *disease incidence in the non-exposed group* and *proportion of exposed patients in the total cohort* both impact the AUC-OR relationship, unlike the RR-OR relationship which is independent of *proportion of exposed patients in the total cohort*.

## Results for Real-World Data

For age alone, in the total cohort (training + test), AUC was 0.78 (continuous) and 0.71 (binary). The optimum classification threshold to maximize Youden's Index (sensitivity + specificity - 1) was 71 years. For LDH alone, in the total cohort, AUC was 0.66 (continuous) and 0.57 (binary). The optimum threshold was 380 U/L. In both cases, we noticed large differences compared to the pre-selected thresholds (65 years and 280 U/L, respectively). With the features combined, in the training set, the models achieved AUC of 0.83 (continuous) and 0.74 (binary). In the test set, the models achieved AUC of 0.84 (continuous) and 0.78 (binary), a statistically significant difference (*p* = 0.006).

## Discussion

The use of OR is a valid starting point when investigating if there is any association between a binary feature, say gender, and a binary outcome, say overall survival after 5 years of cancer treatment. However, the presence of such an association should not be taken to mean that the feature can be used as a predictor for the outcome. In this paper, we performed two experiments, one where the binary feature is common in the cohort (50% patients exposed), and another where the binary feature is relatively rare in the cohort (4.76% patients exposed). To illustrate why the incidence/prevalence of a binary feature in the population has a massive impact on its predictive performance, let us consider two numerical examples.

In example 1 (analogous to Experiment 1), DE = 140, HE = 60, DN = 60, HN = 140. In this case, the exposed group (140+60) and non-exposed group (60+140) both have the same size. The odds ratio is 5.44, and the AUC is 0.7. If we were to build a linear discriminant classifier using just this feature, it would classify all the exposed patients as diseased, and all the non-exposed patients as healthy. Thus, the sensitivity would be 140/(140+60) = 0.7, as would the specificity, balanced accuracy (mean of sensitivity and specificity), PPV, and NPV.

In example 2 (analogous to Experiment 2), DE = 7, HE = 3, DN = 60, HN = 140. In this case, the exposed group (7+3) is 20 times smaller than the non-exposed group (60+140). The odds ratio is still 5.44, but the AUC is only 0.54. If we were to build a linear discriminant classifier using just this feature, it would classify all the exposed patients as diseased, and all the non-exposed patients as healthy. Thus, the sensitivity would be 7/(7+60) = 0.10, the specificity would be 140/(140+3) = 0.98, and the balanced accuracy would be 0.54. The PPV would be 7/(7+3) = 0.7 and the NPV would be 140/(140+60) = 0.7.

If the goal is to use a binary feature for patient stratification, it is essential to mention the predictive performance of such a feature, including the metrics mentioned above. Sensitivity and specificity are intrinsic to the classifier and independent of prevalence. When it is necessary to account for sensitivity/specificity as well as prevalence, PPV and NPV are useful metrics. AUC and balanced accuracy values above 0.8 are routinely achieved for *continuous* predictors, for example tumor volume for predicting the risk of distant metastasis in head and neck cancer (19). For binary predictors, however, such an AUC value is evidently scarce in literature. Hence, it is more realistic that (i) binary predictors may improve the performance of an already existing predictive model (e.g., adding gender to a COVID-19 model that uses blood biomarkers at time of hospital admission to predict risk of severe disease and assist triage) or (ii) several binary predictors may work in conjunction to create a well-performing predictive model. But the discovery of a single binary feature with a large OR (e.g., 5.44, as in the example above) does not necessarily mean that precision medicine is possible using only that particular predictor or biomarker.

When collecting data for a predictive model, whenever possible, it is better to use *ordinal categorical* variables (i.e., a scale) or *continuous* variables rather than convert them to *binary* variables. This statement does not apply to features that are naturally dichotomous (e.g., pregnancy). For example, when considering a pre-existing condition like alcohol abuse, using a scale (say 0–4, tied to the average alcohol consumption per week) is superior to a simple binary feature (alcoholic vs. not alcoholic). When considering a pre-existing condition like obesity, using a continuous variable like body mass index is superior to a simple binary (BMI ≥ 30 vs. BMI < 30). The reason is that the decision threshold of the predictive model can then be tuned based on the training set data, whereas for a binary variable, no such tuning is possible. This has been demonstrated in our Results using real-world COVID-19 data. Nonetheless, we emphasize that our statement is about collecting data, not necessarily about data analysis. If the data analyst wants to use the feature as dichotomous, they may. A feature that has been collected as a continuous or ordinal categorical variable can easily be converted to a binary feature during data analysis. By contrast, if the feature has been collected as dichotomous, it is cumbersome to recover the feature as a continuous or ordinal categorical variable.

## Conclusions

We have presented a simple deterministic simulation to demonstrate the relationships between AUC, RR, and OR. It confirms our hypothesis that in the context of modeling clinical outcome data, presenting only odds ratios of binary predictive features is misleading and should be supplemented by metrics like AUC and balanced accuracy which are needed to understand the predictive performance.

## Author Contributions

AC designed the simulations and examples and wrote the manuscript. HW provided additional machine learning expertise to refine the message of the paper and edited the text for improved clarity. GW was responsible for identifying that odds ratio is a poor metric for predictive performance. PL is the senior author and provided valuable feedback on maximizing the impact of the paper. All authors contributed to the article and approved the submitted version.

## Conflict of Interest

The authors declare that the research was conducted in the absence of any commercial or financial relationships that could be construed as a potential conflict of interest.
